# Seasonal Dynamics and Legionellosis-Associated Hospitalization in Spain: A Retrospective Study

**DOI:** 10.3390/pathogens14050411

**Published:** 2025-04-24

**Authors:** Enrique Gea-Izquierdo, Rossana Ruiz-Urbaez, Valentín Hernández-Barrera, Ángel Gil-de-Miguel

**Affiliations:** 1 Department of Medical Specialties and Public Health, Rey Juan Carlos University, 28922 Madrid, Spain; 2 Faculty of Medicine, Pontifical Catholic University of Ecuador, Quito 170143, Ecuador; 3 María Zambrano Program, European Union, Madrid, Spain; 4 Internal Medicine Service, Eugenio Espejo Hospital, Quito 170403, Ecuador; 5 CIBER of Respiratory Diseases (CIBERES), Instituto de Salud Carlos III, 28029 Madrid, Spain

**Keywords:** legionellosis, seasonality, hospitalization rate, case fatality rate, cost, Spain

## Abstract

Legionellosis is a serious respiratory disease with a high mortality rate, particularly if it is untreated or occurs in the immunocompromised. Legionellosis must be reported in the Spanish Epidemiological Surveillance System. To optimize the epidemiologic knowledge of legionellosis and improve prevention, we have investigated whether the disease is associated with seasonality. This study has described legionellosis cases, the temporal trend by seasonality, hospitalization rate, case fatality rate, and costs by autonomous community and season. We retrospectively reviewed cases of legionellosis, documented patient and clinical characteristics, diagnostics, and seasonality of infection. This study combined national legionellosis notification and hospital discharge data that were linked via the Spanish National Health Service to provide a dataset of hospitalized cases occurring between 2002 and 2021 in Spain. There was a significant increase in the number of legionellosis cases due to the season of the year in Spain. An association between legionellosis and factors related to seasonality is suggested. An increasing trend in case fatality rate, seasonality, and regionality and a decrease in legionellosis hospitalization in Spain were identified. The characterization of changes in legionellosis trend and seasonality and timely synchronization and harmonization of hospitalization records are essential to strengthen disease monitoring and inform potential interventions in an epidemiological way.

## 1. Introduction

Legionella is a waterborne bacterium responsible for legionellosis, which occurs most frequently in susceptible persons, including those of an older age [1], those with chronic diseases or immunosuppression [2], and former or current smokers. The generic term legionellosis is used to describe the two forms of presentation of the disease. Pontiac fever is the non-pneumonic form, which is mild and self-limiting, from which the patient recovers spontaneously in 2 to 5 days. The most serious form is pneumonic Legionnaires’ disease, which has a rapid evolution and is potentially fatal if adequate treatment is not initiated. Therefore, legionellosis varies from a mild flu (Pontiac fever) to an atypical, often severe, form of pneumonia (Legionnaires’ disease) [3]. While approximately 9% of Legionnaires’ disease cases are fatal, mortality associated with health-care-associated Legionnaires’ disease is higher, with reported case fatality rate (CFR) historically being as high as 46% [4].

From the point of view of public health, the relevance of legionellosis is given by its frequent presentation in the form of outbreaks, both community-based and those originating in hospitals and other health and social-health institutions. Legionellosis is a disease with worldwide distribution, with better documentation of incidence in developed countries, where outbreaks of different magnitudes and locations are detected. In Spain, as in most European countries, the reporting of cases of legionellosis has increased significantly due to the increased use of antigen detection in urine, the increase in clinical suspicion of the disease, and the alert in the healthcare system. The incidence in Spain maintained an upward trend until 2019 and fell by 13% in 2020, as has occurred with other diseases. This is an effect attributed to the COVID-19 pandemic, where the limitation of movement and the difficulty of surveillance maintenance and travel may have contributed to some extent. In Spain, the highest mortality rate was observed in men and women who contracted the disease at 65 years of age and older. The fatality rate was 8.4% of cases, lower than that of the European Union average [5].

As mentioned, until the pre-pandemic period, the number of reported cases of legionellosis increased in Spain. Previous studies have investigated the associations between legionellosis incidence and meteorological factors [6], but the Spanish hospital data remained unexplored. In this country, legionellosis affects the following three main areas: community, nosocomial, and travel. That is, cases related to the community, the hospital setting, and those occurring among foreign tourists, reported through the European Surveillance Scheme for Travel-Associated Legionnaires’ Disease (EWGLINET) network and originating from the health authorities of the country of residence of the case, as well as cases of Spaniards traveling within Spain or outside of Spain (reported by the epidemiology services of the autonomous community of residence of the case). However, it is essential to understand the scope of the disease in health terms. Population susceptibility and behaviors are significant, but environmental influences are also important considerations. To describe its potential economic impact, it is necessary to understand the epidemiology of the disease and its national distribution. Therefore, a thorough understanding of hospital-based legionellosis cases is necessary, especially their correspondence with autonomous communities. It is suggested that there may be significant differences in the onset of the disease, the knowledge of which could help us to understand and prevent its behavior based on geographical area. Furthermore, given the knowledge of some of the factors that may influence the incidence of the disease, it is deemed necessary to conduct a detailed study of legionellosis cases based on seasonality [7]. Seasonality exerts a significant influence on the potential occurrence of cases/outbreaks due to climatic conditions that may favor the development of the causative agent, *Legionella* spp. [8]

Seasonality has long been recognized as a characteristic of many human infections, but the mechanisms underlying seasonality, particularly for legionellosis, remain poorly understood. A better understanding of the drivers of seasonality could provide insights into the relationship between the physical environment and infection risk, which is particularly important in the context of global ecological change in general and climate change in particular.

In general terms, seasonality represents an oscillation in the effective reproductive number of pathogens, which, in turn, must reflect oscillatory changes in infectivity, contact patterns, pathogen survival, and host susceptibility. Epidemiological challenges in correctly identifying seasonal risk factors include a lack of adjustment for the predictable correlation between disease incidence and seasonal exposure. The existing evidence suggests that the seasonality of pathogens may be driven by increased pathogen survival during winter, and also by increased host susceptibility resulting from relative “winter immunosuppression” [9].

The objective of this study is to analyze the hospital impact of legionellosis in Spain, considering seasonality, hospitalization rate (HR), CFR, and average costs by autonomous community.

## 2. Methods

### 2.1. Study Design

In this study, we retrospectively analyzed all patients requiring hospitalization with newly diagnosed legionellosis in the Spanish National Health Service over 20 years. The patients with legionellosis were examined according to the autonomous community of correspondence.

### 2.2. Data Source and Study Population

Patients with legionellosis were extracted from the Minimum Basic Data Set (MBDS) of the Spanish Ministry of Health from the Government of Spain. The MBDS database includes diagnosis and procedure codes, which were used to identify relevant conditions, including seasonality. Weights were recorded for each discharge record, which was applied in the analysis to obtain national estimates and disaggregation through autonomous communities.

The hospital discharge report was obtained between January 2002 and December 2021, with a cover hospitalization population of approximately 98%. All patients with a principal diagnosis of legionellosis were included and identified by International Classification of Diseases (ICD), Ninth Edition, Clinical Modification (ICD-9-CM) codes “482.8” and “482.84”, and 10th Revision, ICD-10-CM codes “A48.1” and “A48.2” from 2016 to 2021, to screen for cases. The distribution of the population was analyzed by calculating the global dependency index, and the primary outcome in this study was in-hospital mortality (all-cause death during the period of hospitalization). The secondary outcomes were total hospitalization costs and length of hospitalization stay.

### 2.3. Hospitalization and Demographic Data

The Ministry of Health keeps administrative, demographic, and clinical data of all hospital admissions that cover Spain. To obtain the current list of the categories used in this study, the ICD-9-CM and ICD-10-CM diagnosis codes from both hospital data and medical services data were used. Hospitalization and demographic data were collected from electronic medical records from MBDS. The MBDS data contain only one diagnosis code per record.

All of the cases confirmed legionellosis infection, legionellosis testing, or legionellosis-associated respiratory disease. The principal reason for hospitalization was the primary diagnosis code, and admission was considered a single hospital episode. The annual HR, length of hospital stay, and CFR were calculated using municipal register data [10]. We calculated the estimated fatality rate by dividing the number of fatal cases by the number of the reported incident cases in each year.

Demographic and clinical information were included. For each case, the following specific data were gathered: confirmed diagnosis of legionellosis, year, autonomous community residence, hospital characteristics, season of the event, age (0 to >85), sex, start date contact, date of admission, end date contact, intervention date, contact type, hospital discharge type, length of hospitalization stay, mortality risk, and cost. All of the information was retrieved from the database. The entirely anonymized patients’ data, encoded from medical records by specialized codifiers, using the ICD, were the data source for this study.

The diagnosis type discarded was considered a comorbidity (pre-existing condition) or a complication (a condition arising during the hospital stay). The baseline medical history was retrospectively collected.

### 2.4. Statistical Analysis

Descriptive statistics were calculated to summarize the clinical data. The categorical data were expressed as numbers (%) and continuous data as mean and standard deviation (SD).

Student’s *t*-test was used for comparing continuous variables, while the chi-square test was used to assess differences in proportions. Poisson regression was used to assess differences in HR during the 20-year study period. In all tests, the significance level used was *p* < 0.001, which was considered as statistically significant. The analyses were carried out using Stata software version 16.1.

### 2.5. Ethical Statement

Since de-identified data were used in this study, patient consent to review their medical records was not required.

## 3. Results

**Figure 1:**
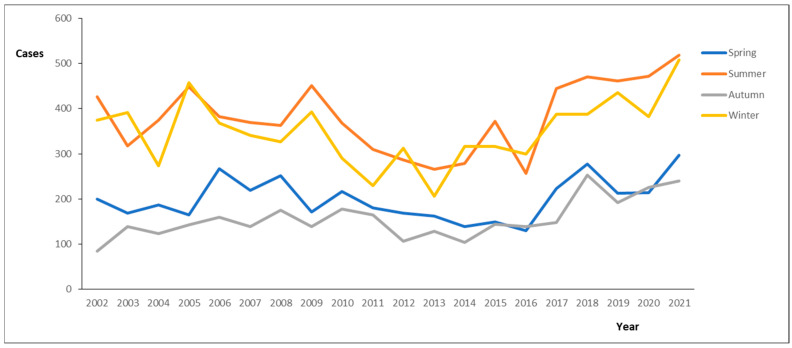
Cases of legionellosis by season and year (2002–2021).

Between 2002 and 2021, 21,300 cases of legionellosis were reported in Spain. In total, there were 10,463 cases of legionellosis with an onset between 1 January 2002 and 31 December 2011; and 10,837 cases reported between 1 January 2002 and 31 December 2011 were included in this study. Among these cases, 74.78% were males, 25.22% females, and 21.2% were aged between 60 and 69 years old. Most cases occurred in the summer months between June and November. However, winter events related to cases of legionellosis were also considered relevant in the studied period (Figure 1). In fact, some years showed higher winter values than summer values, as follows: 2003 (392 cases), 2005 (457 cases), 2012 (312 cases), 2014 (316 cases), and 2016 (300 cases). The total number of cases (2002–2021) by season was as follows: spring (3996), summer (7635), autumn (3120), and winter (6995). The number of cases by time series and season was as follows: 2022–2011 (spring = 1965, summer = 3699, autumn = 1414, winter = 3385) and 2012–2021 (spring = 1937, summer = 3746, autumn = 1657, and winter = 3497).

**Figure 2:**
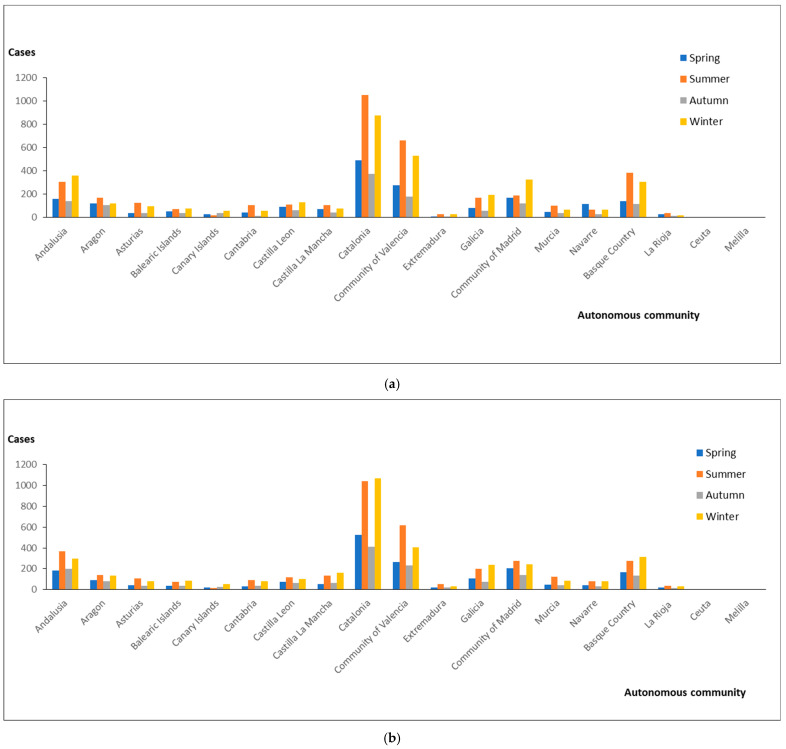
Cases of legionellosis by season and autonomous community: 2002–2011 (**a**) and 2012–2021 (**b**).

Among the 17 autonomous communities and 2 autonomous cities, all reported annual cases from 2002 to 2021 in all seasons, except Ceuta (0 cases in spring and autumn from 2012 to 2021) and Melilla (0 cases in spring and summer from 2002 to 2011) (Figure 2). The highest number of cases per autonomous community was reported in Catalonia and the Community of Valencia. Both showed the highest number of cases in the summer months. This behavior was the same in the two temporal series studied.

**Figure 3:**
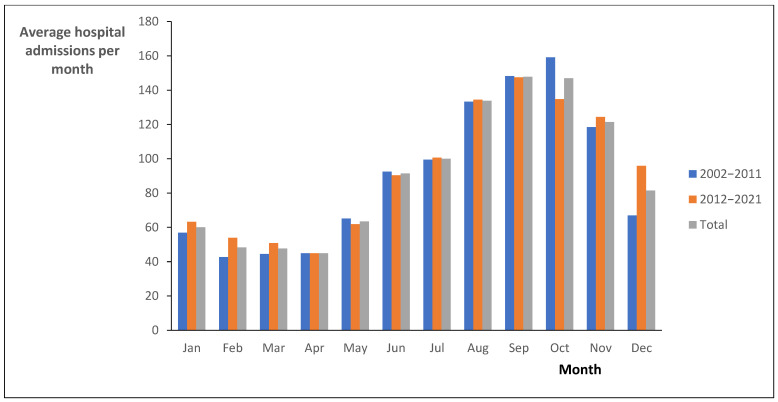
Average hospital admissions for legionellosis per month in Spain, from 2002 to 2011, 2012 to 2021, and 2002 to 2021.

The average hospital admissions per month (Figure 3) showed the highest values between August and November. September reached the total score (2002–2021) of 147.85, while in October in 2002–2011 it was 159.1, and in September in 2012–2021 it was 147.5). An increase in the general trend was observed in the average hospital admissions per month from summer to late autumn.

Depending on seasonality, it is evident that the months of February, March, and April had low average hospital admissions than the rest of the year vs. July–November (broader period of high incidence). Also, considering that cases occur year-round, there are indications that the increase in the number of cases is associated with the increase in temperature/rainfall (summer/winter), while the decrease in the number of cases is expected in the months with lower admissions. Overall, the presence of monthly seasonality was confirmed for all cases, as well as an indication of “positive associations” with some meteorological factors and corresponding legionellosis.

The legionellosis HR and CFR by autonomous community in Spain were calculated, as shown in Table 1. The HR varied from Navarre (4.57) to Ceuta (0.24). The total HR was 2.38 (*p*-value = 0.000) (2002–2021). The CFR varied from Ceuta (50) to Melilla (0). The total CFR was 6.45 (*p*-value = 0.023) (2002–2021).

The average costs (EUR) by autonomous community and season of the year (2002–2011, 2012–2021, and 2002–2021) are shown in Table 2. The highest average cost per season (EUR 7176.14 /autumn) and the lowest per season (EUR 6126.22 /summer) of the entire study corresponded to the 2002–2011 series. There are relevant differences in healthcare costs depending on the autonomous community, the season of the year, and the time series.

Regarding the 20-year period (2002–2021), the highest value was in autumn (7149.92) and the lowest was in summer (6214.89). Large variations in average costs between autonomous communities were observed within a single season, especially in summer. In general, there were notable variations in the average costs of each autonomous community throughout the different seasons.

## 4. Discussion

In this study, we provide a comprehensive overview of the influence of seasonality on the distribution of cases of legionellosis in Spain between 2002 and 2021. The high frequencies of legionellosis reported from 2002 to 2021 showed a seasonal trend. In relation to the effect of seasonality on the frequency of legionellosis, we determined significant differences in the number of admissions in Spain between autonomous communities. However, there were no great differences in the number of patients in the two time series studied and no relevant differences in behavior in each season by years (no fluctuations in seasonal peaks). This approach mitigates the characterization of seasonality that has been typically limited to the description of months with high incidence, which is often inconsistent, imprecise, and lacks the ability to formally compare seasonal variations. In relation to potential confounding variables influencing the observed trends, seasonality and long-term trends themselves can partially contribute to confounding bias. However, it should also be considered that unmeasured/measured time-varying confounders are also responsible creating bias between exposure–disease (legionellosis) relationships. This epidemiological study has demonstrated a strong association between seasonal variations and hospital admissions of legionellosis. Our study has also suggested a seasonality of hospitalization, which is more common in summer and late autumn. In fact, some authors have revealed that legionellosis appears more frequently during summer and autumn rather than at other times of the year in the Northern Hemisphere [11,12]. Others report seasonal cases of legionellosis associated not only with longer summer periods, but also with higher temperatures in spring and autumn [13,14]. Finally, other authors have determined that the disease is more common during late spring and autumn months than at other times of the year. This approach aligns with Southern Hemisphere jurisdictions with a temperate climate, but not in states with a tropical climate [15]. In fact, this is similar to the legionellosis epidemiology in the Northern Hemisphere, but the seasonality appears to be what we have mentioned before, possibly a reflection of recent years of humid and warmer weather [16].

Significant seasonal variations were observed in several communities, with increasing legionellosis cases observed during summer and late autumn/winter. Winter was a critical season because the mechanisms of seasonal influence on the host’s immune system vary, and the immune system weakens during this season. In relation to the autumn epidemic peaks, with the onset of cooler weather, people have increased exposure to the airborne/waterborne pathogen. Furthermore, higher relative humidity can also affect the stability of the airborne droplets in which the pathogens are transmitted, which increases the risk of the causative agent and potential hospitalizations. In fact, Legionella spp. do not link very well to dry environments and are more sensitive than other pathogens to dry conditions. This study supports the hypothesis that wet conditions (autumn/winter) enhance the likelihood of ambient human exposure to biological agents and subsequent infection, contribute to the much larger proportion of sporadic cases, and could increase legionellosis cases. Furthermore, this approach, which promotes legionellosis cases, may also explain the geographic patterns that we observed as lower associations in the dryer autonomous communities. It should be noted that, in spring, when many accommodations reopen after seasonal winter closure, it is very relevant to implement hygienic-preventive maintenance conditions to avoid Legionella contamination [17]. Legionellosis demonstrates distinct seasonality, and temperature is strongly correlated with season, making it difficult to disentangle their independent effects [18].

In Europe, improved epidemiological surveillance has been noted as a potential reason for the continued increase in legionellosis incidence. Over the last years, notification rates of this disease have almost doubled in the European Union/European Environmental Agency, from 1.4 in 2015 to 2.2 cases per 100,000 of the population in 2019 [19]. The seasonal variability of legionellosis incidence is likely influenced by population behavior and susceptibility. Seasonality used to affect a population for more than a year, principally depending on the temperature. This is a relevant factor because it can be associated with an increase in the population of the bacteria’s natural host (which increases due to human activity). Additionally, seasonality can affect cases of legionellosis by increasing the susceptibility of the population to an infection in the medium term and increasing the current transmission rates through increased microbiological concentrations in increased exposure. Of course, hospital admissions could increase. Fischer et al. showed that an increased transmission rate could result in an increased odds ratio during or before the incubation period, when the bacteria start to proliferate in the environment. They also clarify that an increase in susceptibility would likely show a larger odds ratio during the incubation period or in the longer term [6].

In 2021, we noted a pre-seasonal increase in legionellosis compared with the same months in previous years. An increase in legionellosis was identified before the usual peak incidence in late summer/early autumn during the swine influenza (H1N1 strain) outbreak [20,21,22]. However, 2021 was an atypical epidemiological year related to the increased incidence of legionellosis during the pandemic. In addition, legionellosis shows a summer-through-early-fall seasonality that is more evident as incidence increased, which could imply that the cyclical factors causing seasonal patterns (like weather) are becoming more extreme. Based on the increase in pre-seasonal legionellosis, we proposed the aim to study new factors that could explain this increase. The higher incidence of legionellosis in the summer months can be explained by the increased use of air–water exchange systems, which provide suitable environments for the growth and transmission of Legionella. Some authors have considered the potential antecedent/concomitant influenza predisposing to/or presenting with legionellosis (co-infection). They point out that legionellosis often presents as an influenza-like illness and often mimics influenza [23]. Other influencing factors could be related to climatology (i.e., wind, precipitation, temperature, and relative humidity), which may or may not be linked to climate change [23,24]. Some authors have estimated that the increased prevalence of legionellosis may be due to precipitation, being supported by not only a wet and humid environment, but also by precipitation, which is significantly associated with increased rates of legionellosis. Additionally, the risk of legionellosis is significantly associated with increased rainfall 6–10 days before disease onset (rainfall increases organic sediments and contamination with other microbes) [25]. Other authors have found that hospitalizations increased during months with flooding due to extreme storms and were positively associated with monthly precipitation and soil moisture, which are common flood-indicator variables, and that extreme or seasonal flood events are more strongly associated with increased hospitalizations, whereas temperature alone is not [26]. This lag is consistent with a time frame that would be expected from exposure to and infection and incubation of biological agents and sometimes, consequently, hospitalization.

The principal limitation of this study is the broad context, and it is unclear whether similar results would be obtained in other countries or areas. However, in order to design appropriate measures to control the disease, it is important to understand the patterns of incidence in the entire (national) territory. Nevertheless, on-going global warming may possibly affect the seasonality of legionellosis, which may result in an increasing incidence in Spain. If the incidence of legionellosis depends on local weather, the baseline rate of legionellosis might be extremely low year-round in some locations and during specific seasons in other locations. Therefore, it is difficult to determine with certainty that legionellosis increased as a result of the overall effect of seasonality mediated by other factors (temperature, humidity, and ultraviolet radiation). Because of the complex relationship between legionellosis hospitalization and seasonality, the true lag structure is not known. Legionellosis hospitalizations are an underestimation of the true number of cases of Pontiac fever because there were few cases without a corresponding diagnosis for Legionnaires’ disease. Despite these limitations, this study provides prominent findings to deeply understand the behavior and characteristics of legionellosis regarding seasonality.

The present study has a huge strength. In epidemiological terms, the data were collected from patients who came to Spanish hospitals (public and private) within a large area (around 98% of Spain). Additionally, the use of hospitalization ICD codes does not result in misclassification, so this was unlikely to systematically bias our findings. This provides great robustness to this study, and the control of more geographically resolved data eliminates the possibility of misclassification bias.

We estimated the fatality of legionellosis in Spain over 20 years by utilizing a national database that is open to the public. The sensitivity and positive predictive value of the death certificate data were adequate, so the “trend analysis” possibly reflects the entire scene of the disease in Spain. Also, we analyzed the seasonality and regionality of the disease across the country. Through this study, we have revealed an increasing “trend” in CFR, seasonality, and regionality in Spain, but not in HR [10]. Understanding the impact of the seasons on legionellosis supports the interpretation of regional distribution, HR, and CFR. This could help in the prevention of legionellosis and better guide the control of the disease.

Identifying the reasons for the seasonality of legionellosis can offer opportunities for preventive measures and control of the disease and can even assist in the development of effective policies and enable the more efficient and effective use of resources. Moreover, environmental factors can affect pathogen abundance and can be used to improve prevention measures and health training strategies, especially in critical areas. It can be postulated that epidemiology surveillance is driven by seasonal changes directly affecting pathogen abundance and survival, because annual changes in seasonality can be associated with changes in the environmental variables affecting legionellosis. Our results confirm that executing an appropriate maintenance program before and during the period of the seasonal increase in legionellosis [27] could reduce its incidence. In the analysis of legionellosis, we identified that hospitalization cases decreased and CFR increased; however, the HR declined. Additionally, we estimate that warming temperatures and the growing number of ageing people could influence the incidence of the disease on an increasing trend.

Legionellosis is a notifiable disease in Spain and is included as such in the National Epidemiological Surveillance Network [27]. Cases and outbreaks are reported through this network. This allows the collection and information analysis of cases and outbreaks of legionellosis in order to detect problems, assess changes over time and space, and contribute to the implementation of preventive and control measures against this disease.

Legionella infection is generally acquired in the community and nosocomial settings, making it necessary to distinguish between these cases and those associated with travel or those occurring in other settings in epidemiological surveillance. The current state of scientific and technical knowledge, the experience accumulated in the application of regulations, and the results of epidemiological and environmental studies of cases and outbreaks that have occurred in recent years have made it mandatory to consider technical improvements, new risk management measures, and the necessary innovations for greater control of susceptible facilities or equipment [27]. In Spain, the health authority must coordinate the actions of the companies, professionals, entities, and administrations involved in the investigation of cases or outbreaks of legionellosis, taking into account the provisions of the National Epidemiological Surveillance Network. However, it is considered necessary to continue investigating the aspects that lead to the proliferation of Legionella, as well as possible procedures for its effective elimination, adapting regulations accordingly for future advances.

In conclusion, there was a significant increase in the number of legionellosis cases due to the season of the year in Spain. These data suggest an association between causative agents and factors related to seasonality. Legionellosis CFR has risen nationwide for 20 years, and the increase was associated with autonomous community focus and more pronounced seasonality. These factors suggest that deeper research into the effects of weather may further elucidate the rising incidence of the disease. Our study details the increased risk of legionellosis case occurrence linked to seasonality in 2002–2021. Currently, Spain’s summers are setting high temperature records, and this may boost the burden of legionellosis in future years. Future research should aim to disentangle the main drivers of legionellosis occurrence and its impact on public health and should examine the associations among weather conditions and legionellosis throughout Spain, particularly in the south. To this we should add the need for an improvement in detection methods and the quality of reporting to better characterize the seasonal change in pathogen ecology and diminish the fatality of the disease.

The development of predictive strategies for the control of legionellosis requires a better understanding of microbial seasonality, improved reporting of legionellosis, and a detailed analysis of seasonal changes. The accurate characterization of changes in case trends, seasonality, and timely epidemiological records is essential to strengthen disease monitoring and inform potential interventions to prevent the disease.

Improved seasonal recognition, the study of other environmental variables, and the use of hospitalization indicators will allow public health preventive interventions and targeted treatment, and will have the potential to enhance patient outcomes in health systems.

## Figures and Tables

**Table 1:** Legionellosis hospitalization rate (HR) (a) and case fatality rate (CFR) (b) by autonomous community in Spain (2002–2011, 2012–2021, and 2002–2021).

a				
Autonomous Community	HR			
	2002–2011	2012–2021	2002–2021	*p*-Value *
Andalusia	1.21 (1.13–1.29)	1.24 (1.16–1.32)	1.23 (1.18–1.28)	0.000
Aragon *	3.96 (3.62–4.3)	3.39 (3.08–3.7)	3.67 (3.44–3.9)	0.000
Asturias	2.81 (2.49–3.13)	2.55 (2.24–2.86)	2.68 (2.46–2.9)	0.000
Balearic Islands	2.48 (2.17–2.79)	2.04 (1.78–2.3)	2.24 (2.04–2.44)	0.000
Canary Islands	0.72 (0.6–0.84)	0.53 (0.43–0.63)	0.62 (0.54–0.7)	0.000
Cantabria	3.89 (3.38–4.4)	4.14 (3.62–4.66)	4.02 (3.65–4.39)	0.000
Castilla Leon	1.57 (1.41–1.73)	1.47 (1.32–1.62)	1.52 (1.41–1.63)	0.000
Castilla La Mancha *	1.53 (1.36–1.7)	2.03 (1.84–2.22)	1.78 (1.65–1.91)	0.000
Catalonia *	3.93 (3.78–4.08)	4.06 (3.92–4.2)	3.99 (3.89–4.09)	0.000
Community of Valencia *	3.48 (3.31–3.65)	3.06 (2.91–3.21)	3.27 (3.16–3.38)	0.000
Extremadura *	0.67 (0.52–0.82)	1.14 (0.94–1.34)	0.91 (0.78–1.04)	0.000
Galicia *	1.84 (1.68–2)	2.27 (2.09–2.45)	2.06 (1.94–2.18)	0.000
Community of Madrid	1.32 (1.23–1.41)	1.32 (1.23–1.41)	1.32 (1.26–1.38)	0.000
Murcia *	1.84 (1.61–2.07)	2.06 (1.83–2.29)	1.96 (1.8–2.12)	0.000
Navarre *	4.57 (4.03–5.11)	3.57 (3.11–4.03)	4.06 (3.71–4.41)	0.000
Basque Country	4.4 (4.12–4.68)	4.09 (3.82–4.36)	4.24 (4.05–4.43)	0.000
La Rioja	3.14 (2.51–3.77)	3.53 (2.87–4.19)	3.33 (2.88–3.78)	0.000
Ceuta *	1.6 (0.7–2.5)	0.24 (0–0.57)	0.88 (0.42–1.34)	0.000
Melilla	0.28 (0–0.67)	0.48 (0.01–0.95)	0.39 (0.08–0.7)	0.999
Total *	2.41 (2.36–2.46)	2.36 (2.32–2.4)	2.38 (2.35–2.41)	0.000
**b**				
**Autonomous Community**	**CFR**			
	**2002–2011**	**2012–2021**	**2002–2021**	*p*-Value *
Andalusia *	8.83 (7.04–10.62)	11.57 (9.63–13.51)	10.25 (8.92–11.58)	0.452
Aragon	10.37 (7.73–13.01)	10.69 (7.83–13.55)	10.52 (8.58–12.46)	0.086
Asturias	7.33 (4.38–10.28)	4.92 (2.31–7.53)	6.21 (4.22–8.2)	0.621
Balearic Islands	8.91 (5.36–12.46)	7.63 (4.24–11.02)	8.28 (5.82–10.74)	0.837
Canary Islands	7.91 (3.42–12.4)	7.83 (2.92–12.74)	7.87 (4.56–11.18)	0.821
Cantabria	5.88 (2.78–8.98)	2.48 (0.52–4.44)	4.1 (2.29–5.91)	0.652
Castilla Leon *	10.43 (7.41–13.45)	6.41 (3.88–8.94)	8.51 (6.52–10.5)	0.444
Castilla La Mancha	5.69 (3.06–8.32)	7.69 (5.13–10.25)	6.85 (5–8.7)	0.305
Catalonia *	4.52 (3.75–5.29)	5.98 (5.14–6.82)	5.28 (4.71–5.85)	0.003
Community of Valencia	6.75 (5.54–7.96)	6.89 (5.62–8.16)	6.82 (5.94–7.7)	0.016
Extremadura	9.59 (2.84–16.34)	10.57 (5.14–16)	10.2 (5.96–14.44)	0.280
Galicia *	6.93 (4.71–9.15)	2.91 (1.58–4.24)	4.72 (3.48–5.96)	0.717
Community of Madrid	5.76 (4.14–7.38)	6.86 (5.17–8.55)	6.33 (5.16–7.5)	0.914
Murcia	5.56 (2.73–8.39)	7.21 (4.31–10.11)	6.46 (4.42–8.5)	0.706
Navarre *	2.54 (0.68–4.4)	7.83 (4.36–11.3)	4.94 (3.05–6.83)	0.463
Basque Country	3.61 (2.42–4.8)	4.05 (2.75–5.35)	3.82 (2.94–4.7)	0.350
La Rioja	5.21 (0.76–9.66)	3.6 (0.13–7.07)	4.35 (1.57–7.13)	0.854
Ceuta *	8.33 (0–23.97)	50 (0–119.3)	14.29 (0–32.62)	-
Melilla	0 (0–0)	0 (0–0)	0 (0–0)	-
Total	6.24 (5.78–6.7)	6.66 (6.19–7.13)	6.45 (6.12–6.78)	0.023

* Significative difference by year (*p* < 0.001).

**Table 2:** Average costs (EUR) by autonomous community and season of the year (2002–2011, 2012–2021, and 2002–2021).

Autonomous Community	2002–2011								2012–2021								2002–2021							
	Spring		Summer		Autumn		Winter		Spring		Summer		Autumn		Winter		Spring		Summer		Autumn		Winter	
	$\bar{x}$	SD	$\bar{x}$	SD	$\bar{x}$	SD	$\bar{x}$	SD	$\bar{x}$	SD	$\bar{x}$	SD	$\bar{x}$	SD	$\bar{x}$	SD	$\bar{x}$	SD	$\bar{x}$	SD	$\bar{x}$	SD	$\bar{x}$	SD
Andalusia	6863.55	7639.86	6950.29	10,257.30	7884.83	11,387.88	6512.83	6931.17	7024.87	9717.13	5840.44	5936.25	6591.31	8773.90	7985.20	11,219.31	6948.69	8785.96	6337.63	8172.12	7117.04	9924.15	7189.09	9175.91
Aragon	7137.69	7365.50	6786.12	10,742.30	6612.23	7788.30	5995.55	6782.63	6139.52	5976.33	5730.94	6538.50	7971.55	11,274.77	6339.36	9561.66	6688.28	6778.19	6300.14	9054.03	7178.61	9393.96	6176.36	8345.68
Asturias	6004.58	7934.63	5132.42	3959.83	7318.60	9746.68	5337.82	5566.95	4237.12	1112.11	4760.96	3413.01	4668.11	1724.88	4840.15	3526.10	5083.51	5577.38	4958.00	3709.79	6081.71	7294.21	5111.61	4743.23
Balearic Islands	7403.67	8994.24	7747.10	8281.04	8933.13	12,741.53	8601.52	9776.59	5115.72	2536.53	6934.16	10,059.61	6854.80	7736.83	7409.57	10,628.51	6429.59	7078.54	7345.30	9185.46	7867.99	10,463.83	7980.86	10,216.24
Canary Islands	7975.93	9179.18	10,295.18	13,664.18	10,677.86	13,228.09	7508.96	15,121.32	9356.05	14,673.66	11,645.10	22,034.75	6134.60	5849.39	10,778.36	20,296.88	8651.91	12,074.23	10,970.14	18,082.73	8702.53	10,840.82	9085.28	17,799.24
Cantabria	10,916.24	20,497.84	6407.59	8676.71	7520.81	6230.67	5364.35	3648.98	5123.59	2997.51	6986.77	14,676.18	5738.44	7124.63	5037.85	2930.43	8349.88	15,618.26	6677.50	11,829.14	6266.55	6862.83	5174.09	3240.99
Castilla Leon	8972.97	12,154.25	6781.55	9655.27	10,881.70	15,002.35	6682.21	10,567.59	8259.99	10,468.85	7174.45	13,284.04	7012.31	9958.71	6060.73	7605.74	8673.85	11,451.10	6975.55	11,567.26	8842.02	12,695.49	6412.00	9381.45
Castilla La Mancha	7849.45	10,782.10	5895.38	5205.12	7983.51	10,946.75	6072.76	3658.39	5203.37	3319.64	6549.02	8954.94	6606.21	5855.10	6235.68	7968.31	6668.89	8402.23	6262.56	7538.25	7154.46	8243.41	6186.19	6939.39
Catalonia	6290.79	6633.25	5841.48	7349.12	6918.48	8571.28	6095.21	8219.99	7081.54	10,594.03	6269.66	8695.07	7523.11	10,847.98	6647.42	9785.86	6693.70	8879.70	6054.57	8048.12	7234.83	9827.08	6397.76	9113.22
Community of Valencia	6558.39	11,366.23	5533.92	7108.07	6389.87	9786.04	5928.45	8884.88	7668.51	12,720.18	6567.80	10,875.39	6923.09	9845.09	6039.69	8900.37	7082.56	12,026.39	6013.09	9062.25	6675.75	9809.89	5975.53	8886.96
Extremadura	4754.15	1449.05	4231.98	1561.15	7849.51	10,652.66	11,654.14	23,320.37	6095.52	4808.75	5180.81	2640.92	10,435.71	18,528.66	4870.77	1748.59	5706.09	4138.83	4860.43	2365.20	9522.94	16,058.36	8011.22	16,110.20
Galicia	5270.73	3250.48	7187.84	10,622.49	6079.52	3514.93	5746.85	5353.33	6404.91	8876.73	6207.69	8706.00	6869.04	10,067.71	5500.07	4749.77	5919.70	7050.50	6666.97	9650.98	6531.54	7942.12	5608.43	5019.26
Community of Madrid	7117.93	9690.96	6713.52	8607.71	7217.97	8450.72	7275.33	9713.59	8702.07	14,545.18	7235.56	10,446.57	8842.34	12,356.84	8502.46	13,359.02	7968.67	12,543.04	7027.45	9748.22	8108.55	10,780.20	7802.17	11,427.25
Murcia	4477.66	1440.11	6170.58	7890.75	6716.33	9004.18	4501.92	1523.15	5326.59	3437.08	5333.36	5227.01	6490.12	6971.41	5926.46	5411.40	4942.34	2741.63	5714.24	6572.13	6595.14	7930.69	5301.50	4226.73
Navarre	4451.36	2593.88	5824.42	9186.62	4226.43	1091.62	4748.76	2917.44	6964.02	10,378.17	7314.94	14,836.94	8214.14	14,330.44	5632.46	7169.13	5110.16	5825.35	6633.01	12,554.78	6449.09	10,837.60	5230.78	5649.34
Basque Country	6311.50	7963.24	6155.51	7724.76	6315.65	8596.43	5719.06	5658.23	6046.02	10,315.16	5814.94	8862.04	5828.48	7194.05	5909.36	7555.49	6166.62	9307.00	6010.51	8223.43	6048.37	7845.94	5816.04	6687.97
La Rioja	4582.55	1419.94	4401.33	1193.68	6457.55	4993.25	5784.14	4809.92	4477.97	1918.80	5221.72	5116.69	8740.28	17,198.15	4527.23	989.08	4532.44	1660.04	4822.32	3757.54	7856.65	13,707.13	5010.66	3097.57
Ceuta	3959.35	587.26	6349.93	1156.85	5721.18	1965.59	6578.31	4828.28			5054.09				5497.51	1284.32	3959.35	587.26	5917.98	1108.55	5721.18	1965.59	6218.04	3824.75
Melilla	13,255.49				4174.19		12,502.50		4874.01		3919.85		4524.88	748.42	5490.88	1791.97	9064.75	5926.60	3919.85		4407.98	566.62	7243.78	3798.88
Total	6620.07	8782.21	6126.22	8089.84	7176.14	9525.28	6221.14	8162.09	6945.08	10,430.73	6303.21	9339.47	7127.43	10,049.91	6559.21	9415.94	6780.38	9630.82	6214.89	8738.13	7149.92	9809.56	6392.76	8821.90

$\bar{x}$: Average. SD: Standard deviation.

## Data Availability

The datasets analyzed in the current study are publicly available in the Hospital Discharge Records in the Spanish National Health System (MBDS) repository at https://www.mscbs.gob.es/en/estadEstudios/estadisticas/cmbdhome.htm (accessed on 26 January 2024). The information contained in this repository can be accessed without the need for any administrative permissions.

## Abbreviations

The following abbreviations are used in this manuscript:

CFR	Case fatality rate
HR	Hospitalization rate
ICD	International Classification of Diseases
ICD-9-CM	International Classification of Diseases 9th Revision
ICD-10-ES	International Classification of Diseases 10th Revision
MBDS	Minimum Basic Data Set
